# The Clinical Laboratory

**Published:** 1913-02-01

**Authors:** 


### THE CLINICAL LABORATORY.

### II. Diseases Due to Bacterial Infections.*

The general infections which concern a general hospital are typhoid fever, influenza, cerebro-spinal fever, pneumonia, diphtheria, erysipelas, septicæmia and pyæmia, including puerperal fever, rheumatic fever, bacillary dysentery and infantile diarrhœas of infectious origin, anthrax, tetanus, glanders, actinomycosis, gonorrhœa, and tubercle. The bacteriology of some of these conditions is still in a very chaotic state, and therefore the clinical laboratory cannot hope to be of much use in the diagnosis.

### The Agglutination Test.

Typhoid fever cases are still admitted into many hospitals even when recognised, but the chief value of the clinical laboratory is as an aid to diagnosis. The first aid to diagnosis is a leukocyte count. Typhoid is one of the few fevers which show leukopenia instead of leukocytosis, and therefore the demonstration that the patient has one or the other has a very high value as a diagnostic measure. The leukopenia can be demonstrated before the agglutination reaction. A leukocytosis is therefore exceedingly strong evidence against the diagnosis of typhoid. A leukopenia suggests typhoid, but does not necessarily indicate the disease, as some severe infections may show a similar count. The stained film also gives valuable indications as in the early stages of typhoid the eosinophiles often vanish entirely. A normal count for eosinophiles is very strong evidence against typhoid. At the end of the first week one can seek for the agglutination reaction. In most towns this is performed free of charge by the public health laboratories, and, therefore, few clinical laboratories will have to be in a position to do the test. As ordinarily performed in this country it requires either a fresh living culture or a killed culture. In Germany, in situations where it may be necessary to perform the test at rare intervals, it seems to be the custom to rely upon "Ficker's Typhus Diagnosticum" (obtainable from Merck). The reaction is a precipitin reaction, and is practically equivalent to the agglutination test. It certainly seems to be worth a place in isolated laboratories where the reaction must occasionally be performed. A leukocytosis is useful in confirming the diagnosis of many complications, and the presence of occult melæna is said to forecast hæmorrhage.

*Influenza* is full of difficulty. At present influenza might be defined as the last refuge of the puzzled physician. Bacteriological diagnosis should be attempted, but is difficult. The mouth should be rinsed out with saline, and the sputum expectorated into a sterile vessel and films spread immediately. These should be stained by Gram's method. The *B. influenza* is a very small rod with rounded ends, which often show polar staining. It is also often intracellular. It should be present in very large numbers before a diagnosis is framed, and tubercle bacilli should be absent. This is all very simple, but the difficulty consists in the differentiation from the different forms of *B. catarrhalis*, which are found in common colds. For this difficult task requiring special media Martin's article in the *Journal of Pathology and Bacteriology* should be consulted.

Cerebro-spinal fever will be discussed under the heading of Nervous Diseases.

The bacteriology of pneumonia is apparently easy, for the organism is easily recognised in the stained films of sputum. The practical difficulties are due to the fact that all gram-positive diplococci are not pneumococci, and that the organism occurs in the saliva of healthy persons.

The sputum should be examined with the precautions mentioned under influenza, the organisms must show the typical shape and capsule, and be present in very large numbers. Before attempting to diagnose pneumonia in a doubtful case one must stain for the tubercle bacillus and make certain that it is absent. In a fair number of cases of pneumonia the organism can be isolated from the blood during life. In making a blood culture one must: (1) Avoid contamination from the skin by scrubbing the skin with 70 per cent. alcohol, and then take the blood direct from a vein by means of a large serum syringe. (2) At least 10 c.c. of blood should be taken, and it should immediately be mixed with a large quantity of broth, in order (3) to avoid the bactericidal effect of the serum. It is advisable to use a six-ounce flask (a medicine bottle will do). This should be examined at the end of twenty-four hours, and if negative at the end of forty-eight. A leukocyte count is of value in some cases (diagnosis from typhoid fever).

### A Good Instance of Bacteriological Diagnosis.

Diphtheria affords one of the best examples of bacteriological diagnosis. The membrane should be torn off with forceps and then smeared over Loeffler's blood-serum media. In cases where there is no membrane a sterile swab of cotton wool is rubbed on the fauces (no antiseptic gargles must be used for several hours previously). The direct examination of the smear by microscopical methods alone is not reliable, save in the hands of an expert, and then negative results must not be accepted. After twelve hours the culture is transferred to a cover slip, fixed by heat and then inverted on to a small drop of Loeffler's methylene blue and examined in water. The only difficulty remaining is to discover whether the organisms with the characteristic appearance are true diphtheria bacilli or pseudo-forms. This is a question for the expert. If the novice is in doubt the result should be returned as true diphtheria and the patient treated as such. When the problem is the discovery of a carrier this way out of the difficulty is illegitimate. The matter should only be trusted to an acknowledged expert.

In erysipelas the streptococci are easily found in films spread from the serum of a blister and stained with Gram. The clinical diagnosis is practically always sufficient.

A Hopelessly Unscientific Procedure.

In septicæmia and pyæmia it is important to demonstrate the organism. It may be found in the blood in some cases, but in others the primary focus must be investigated. In puerperal cases the uterus should be examined. Vaginal contamination can be avoided by using Kelly's urethral speculum introduced into the cervix. In others the organism may be found in the urine, but all urinary findings must be looked upon with suspicion. The pus from a secondary abscess may be useful. Of late years it has become somewhat fashionable in all doubtful cases of septicæmia to cultivate organisms from the pyorrhœa which may be present, and then prepare vaccines for treatment. As many cases of cryptogenic sepsis recover naturally this has been a fruitful source of success to the vaccine therapists. The whole procedure is hopelessly unscientific, although it masquerades as a specially " scientific " branch of medicine.

Malignant endocarditis is really a form of septicæmia and should be investigated in the same manner, especially by blood culture. It is important to remember that some of these cases show a leukopenia, and that therefore the absence of leukocytosis is not of much diagnostic value.

Rheumatic fever is, perhaps unfortunately for the study of the disease, easily recognised from the clinical phenomena. The organism cannot, with the rarest exceptions, be isolated from the blood during life. It is still doubtful whether we are entitled to speak of the rheumococcus or micrococcus rheumaticus as the organism of rheumatic fever. According to Beattie and his followers it is never found in the blood, but is only found in the valvular lesions and the synovial membranes of the joints (*not* in the synovial fluid). It is easily isolated after death. The joint should be opened aseptically and a piece of the reddened membrane cut out and dropped into a milk-broth media. It can be isolated from the synovial membrane weeks after the last appearance of acute joint symptoms. The only way in which the streptococcus rheumaticus can be differentiated from the other streptococci is by injection into the rabbit, in which it causes a typical arthritis. Some pathologists claim that it is merely an intestinal streptococcus which wanders into the tissues during the agonal invasion of the body by the organisms from the alimentary canal. This objection appears to have been successfully met by some recent work of Beattie and Yates. None of the serological tests has yet been applied successfully.

Bacillary dysentery and infantile diarrhœa form a most interesting subject for research. Many of them are apparently due to different strains of bacilli closely related to the colon and dysentery bacilli. Their isolation and identification is one of the most difficult tasks of modern bacteriology. Large quantities of media, some of it very expensive, an immense amount of time, and considerable experience are required. Such work requires all the facilities of a large pathological laboratory.

A Dangerous Laboratory Organism.

In the diagnosis of anthrax the clinical laboratory plays an important part, as the identification of the organism by means of smears stained with Gram's method leads to a diagnosis and appropriate treatment. In tetanus the assistance of the clinical laboratory is not required unless it were for the purpose of tracing contamination to catgut. In glanders of man a bacteriological examination is always required to confirm the clinical diagnosis. Vaccine treatment is said to be of great use, and one can quite imagine that this would be the case in a chronic case. It is a dangerous laboratory organism, for one worker who successfully treated a patient with vaccine was accidentally inoculated in a laboratory and died.

Actinomycosis can often be diagnosed from clinical data, but the examination of the sulphur bodies should be carried out to confirm the diagnosis. This is of importance, for to do good with iodine it must be pushed, and this is not justifiable without a positive diagnosis.

In gonorrhœa a bacteriological diagnosis should always be made where there is the slightest doubt or dispute. This is particularly the case in the female. Some of the pus from the urethra in the male, from the cervix uteri in the female, should be made into smear preparations and stained by Gram. A positive result should be recorded only when one or more leukocytes are found crowded with the typical biscuit-shaped, Gram-negative diplococci. In the male the secretion expressed from the prostate by massage through the rectum may be examined in chronic cases. The organism has also been found in the blood of acute cases of gonococcal septicæmia. The problem of the specific diagnosis of tubercle is one of the most important in clinical medicine, and will be reserved for a special article.

* The first article of this series appeared in our issue of November 23, 1912.

